# 4-Phenyl-1,2,4-tri­aza­spiro­[4.6]undec-1-ene-3-thione

**DOI:** 10.1107/S1600536814009817

**Published:** 2014-05-10

**Authors:** Shaaban K. Mohamed, Joel T. Mague, Mehmet Akkurt, Alaa A. Hassan, Mustafa R. Albayati

**Affiliations:** a Chemistry and Environmental Division, Manchester Metropolitan University, Manchester M1 5GD, England; b Chemistry Department, Faculty of Science, Mini University, 61519 El-Minia, Egypt; c Department of Chemistry, Tulane University, New Orleans, LA 70118, USA; d Department of Physics, Faculty of Sciences, Erciyes University, 38039 Kayseri, Turkey; e Kirkuk University, College of Science, Department of Chemistry, Kirkuk, Iraq

## Abstract

In the title compound, C_14_H_17_N_3_S, the plane of the phenyl ring makes a dihedral angle of 74.90 (4)° with that of the tri­aza­thione ring (r.m.s. deviation = 0.001 Å), while the seven-membered ring adopts a twist-chair conformation. No specific intermolecular interactions are discerned in the crystal packing.

## Related literature   

For various pharmaceutical properties of spiro compounds, see: Chin *et al.* (2008 ▶); Thadhaney *et al.* (2010 ▶). For industrial uses of heterocyclic spiro compounds, see: Sarma *et al.* (2010 ▶). For the crystal structures of two similar compounds, see: Akkurt *et al.* (2013 ▶); Mague *et al.* (2014 ▶). For ring-puckering parameters, see: Cremer & Pople (1975 ▶).
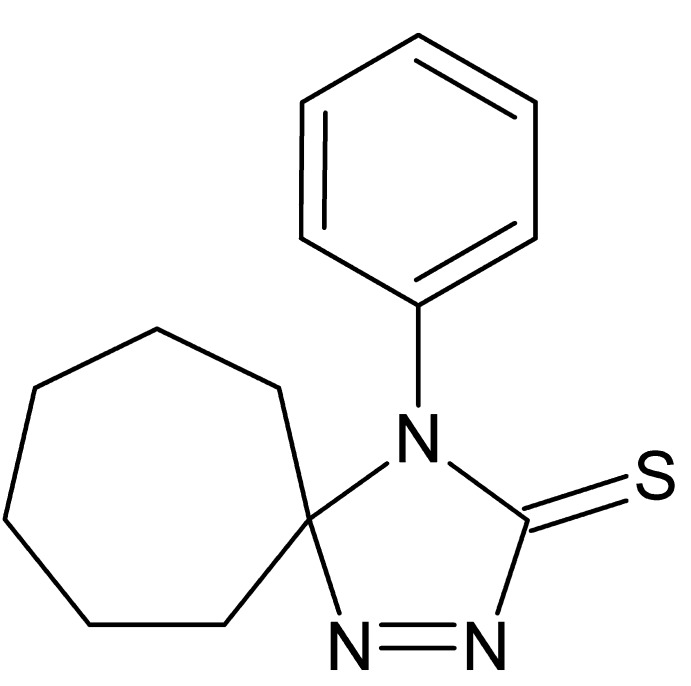


## Experimental   

### 

#### Crystal data  

C_14_H_17_N_3_S
*M* *_r_* = 259.36
Triclinic, 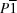

*a* = 9.0578 (5) Å
*b* = 9.1324 (5) Å
*c* = 9.4637 (5) Å
α = 88.2940 (8)°
β = 79.0690 (7)°
γ = 61.6640 (6)°
*V* = 674.89 (6) Å^3^
*Z* = 2
Mo *K*α radiation
μ = 0.23 mm^−1^
*T* = 150 K
0.28 × 0.23 × 0.06 mm

#### Data collection  

Bruker SMART APEX CCD diffractometer
Absorption correction: multi-scan (*SADABS*; Bruker, 2013 ▶) *T*_min_ = 0.85, *T*_max_ = 0.98
12510 measured reflections
3508 independent reflections
3125 reflections with *I* > 2σ(*I*)
*R*_int_ = 0.032

#### Refinement  

*R*[*F* ^2^ > 2σ(*F* ^2^)] = 0.036
*wR*(*F* ^2^) = 0.096
*S* = 1.04
3508 reflections
163 parameters
H-atom parameters constrained
Δρ_max_ = 0.44 e Å^−3^
Δρ_min_ = −0.20 e Å^−3^

### 

Data collection: *APEX2* (Bruker, 2013 ▶); cell refinement: *SAINT* (Bruker, 2013 ▶); data reduction: *SAINT*; program(s) used to solve structure: *SHELXT* (Bruker, 2013 ▶); program(s) used to refine structure: *SHELXL2014* (Sheldrick, 2008 ▶); molecular graphics: *DIAMOND* (Brandenburg & Putz, 2012 ▶); software used to prepare material for publication: *SHELXTL* (Sheldrick, 2008 ▶).

## Supplementary Material

Crystal structure: contains datablock(s) global, I. DOI: 10.1107/S1600536814009817/hg5394sup1.cif

Structure factors: contains datablock(s) I. DOI: 10.1107/S1600536814009817/hg5394Isup2.hkl

Supporting information file. DOI: 10.1107/S1600536814009817/hg5394Isup3.cml

CCDC reference: 1000439

Additional supporting information:  crystallographic information; 3D view; checkCIF report

## Figures and Tables

**Fig. 1.**
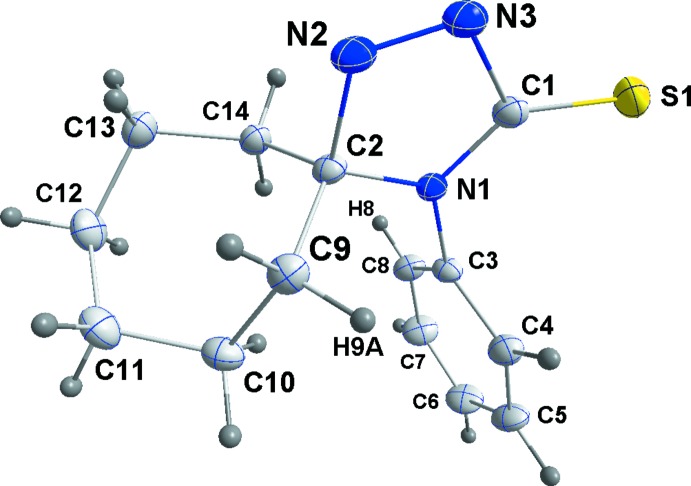
Title compound with 50% probability displacement ellipsoids for non-H atoms.

## supplementary crystallographic information

### 1. Comment

Spiro-compounds are a significant class of of organic compounds due to their wide spectrum of pharmaceutical and applied chemistry aspects. They showed very promising biological activities such as anticancer agents (Chin *et al.*, 2008) and antimicrobial agents (Thadhaney *et al.*, 2010). Some spiro-compounds have also been recently used as antioxidants (Sarma *et al.*, 2010). In this context and as part of our on-going study in synthesis of spiro-compounds for the purpose of biological potential, we report in this study the synthesis and crystal structure determination of the title compound.

In the title compound (I, Fig. 1), a Cremer-Pople analysis of the conformation of the 7-membered ring (C2/C9/C10–C14) gave puckering parameters Q(2) = 0.5606 (14) Å, Q(3) = 0.6549 (15) Å, φ(2) = 272.80 (15)° and φ(3) = 272.01 (12)° (Cremer & Pople, 1975). The total puckerin amplitude is 0.8620 (14) Å.

The phenyl ring (C3–C8) makes a dihedral angle of 74.90 (4)° with the triazathione ring (C1/C2/N1–N3). All bond lengths and bond angles in (I) are comparable with those for the similar compounds that we have reported previously (Akkurt *et al.*, 2013; Mague *et al.*, 2014).

### 2. Experimental

A mixture of 1 mmol (261 mg) of cycloheptan-1-one *N*-phenylthiosemicarbazone and 1 mmol (246 mg) of 2,3,5,6-tetrachloro-1,4-benzoquinone (DDQ) in 30 ml of ethyl acetate was stirred at room temperature. The reaction was monitored by TLC until completion. The precipitated DDQ-H_2_ was filtered off and the filtrate was concentrated by slow evaporation in air to afford the corresponding product. The crude product was recrystallized from ethanol to furnish orange block crystals suitable for X-ray diffraction.

### 3. Refinement

H-atoms attached to carbon were placed in calculated positions (C—H = 0.95 - 0.99 Å). All were included as riding contributions with isotropic displacement parameters 1.2 times those of the attached atoms.

### Crystal data

**Table:**

C_14_H_17_N_3_S	*Z* = 2
*M**_r_* = 259.36	*F*(000) = 276
Triclinic, *P*1	*D*_x_ = 1.276 Mg m^−^^3^
Hall symbol: -P 1	Mo *K*α radiation, λ = 0.71073 Å
*a* = 9.0578 (5) Å	Cell parameters from 9063 reflections
*b* = 9.1324 (5) Å	θ = 2.2–29.1°
*c* = 9.4637 (5) Å	µ = 0.23 mm^−^^1^
α = 88.2940 (8)°	*T* = 150 K
β = 79.0690 (7)°	Plate, orange
γ = 61.6640 (6)°	0.28 × 0.23 × 0.06 mm
*V* = 674.89 (6) Å^3^	

### Data collection

**Table:**

Bruker SMART APEX CCD diffractometer	3508 independent reflections
Radiation source: fine-focus sealed tube	3125 reflections with *I* > 2σ(*I*)
Graphite monochromator	*R*_int_ = 0.032
Detector resolution: 8.3660 pixels mm^-1^	θ_max_ = 29.1°, θ_min_ = 2.2°
φ and ω scans	*h* = −12→12
Absorption correction: multi-scan (*SADABS*; Bruker, 2013)	*k* = −12→12
*T*_min_ = 0.85, *T*_max_ = 0.98	*l* = −12→12
12510 measured reflections	

### Refinement

**Table:**

Refinement on *F*^2^	Primary atom site location: structure-invariant direct methods
Least-squares matrix: full	Secondary atom site location: difference Fourier map
*R*[*F*^2^ > 2σ(*F*^2^)] = 0.036	Hydrogen site location: inferred from neighbouring sites
*wR*(*F*^2^) = 0.096	H-atom parameters constrained
*S* = 1.04	*w* = 1/[σ^2^(*F*_o_^2^) + (0.0469*P*)^2^ + 0.2315*P*] where *P* = (*F*_o_^2^ + 2*F*_c_^2^)/3
3508 reflections	(Δ/σ)_max_ < 0.001
163 parameters	Δρ_max_ = 0.44 e Å^−^^3^
0 restraints	Δρ_min_ = −0.20 e Å^−^^3^

### Special details

**Table:**

Geometry. Bond distances, angles *etc*. have been calculated using the rounded fractional coordinates. All su's are estimated from the variances of the (full) variance-covariance matrix. The cell e.s.d.'s are taken into account in the estimation of distances, angles and torsion angles
Refinement. Refinement on *F*^2^ for ALL reflections except those flagged by the user for potential systematic errors. Weighted *R*-factors *wR* and all goodnesses of fit *S* are based on *F*^2^, conventional *R*-factors *R* are based on *F*, with *F* set to zero for negative *F*^2^. The observed criterion of *F*^2^ > σ(*F*^2^) is used only for calculating -*R*-factor-obs *etc*. and is not relevant to the choice of reflections for refinement. *R*-factors based on *F*^2^ are statistically about twice as large as those based on *F*, and *R*-factors based on ALL data will be even larger.

### Fractional atomic coordinates and isotropic or equivalent isotropic displacement parameters (Å^2^)

**Table:**

	*x*	*y*	*z*	*U*_iso_*/*U*_eq_	
S1	1.18135 (4)	0.44697 (4)	0.80140 (3)	0.0246 (1)	
N1	0.85249 (12)	0.58855 (11)	0.76728 (10)	0.0161 (2)	
N2	0.75975 (13)	0.84504 (12)	0.87544 (11)	0.0222 (3)	
N3	0.91495 (13)	0.75963 (13)	0.87953 (11)	0.0223 (3)	
C1	0.98219 (14)	0.59098 (14)	0.81285 (12)	0.0180 (3)	
C2	0.69598 (14)	0.75078 (13)	0.80402 (12)	0.0169 (3)	
C3	0.85298 (13)	0.44117 (13)	0.71782 (12)	0.0165 (3)	
C4	0.85413 (15)	0.32566 (14)	0.81669 (13)	0.0205 (3)	
C5	0.84438 (16)	0.18734 (15)	0.77211 (14)	0.0255 (3)	
C6	0.83537 (18)	0.16576 (16)	0.63011 (15)	0.0292 (4)	
C7	0.83794 (18)	0.28004 (17)	0.53131 (14)	0.0294 (4)	
C8	0.84735 (15)	0.41916 (15)	0.57489 (12)	0.0219 (3)	
C9	0.55281 (15)	0.73871 (14)	0.91260 (12)	0.0202 (3)	
C10	0.42376 (16)	0.71324 (16)	0.84612 (13)	0.0238 (3)	
C11	0.27415 (16)	0.87693 (17)	0.81639 (14)	0.0267 (3)	
C12	0.31779 (16)	0.96808 (17)	0.69115 (14)	0.0273 (3)	
C13	0.46872 (15)	0.99887 (15)	0.69690 (13)	0.0232 (3)	
C14	0.64134 (14)	0.83880 (14)	0.66771 (12)	0.0192 (3)	
H4	0.86150	0.34080	0.91350	0.0250*	
H5	0.84390	0.10770	0.83880	0.0310*	
H6	0.82740	0.07180	0.60030	0.0350*	
H7	0.83330	0.26350	0.43400	0.0350*	
H8	0.84990	0.49780	0.50780	0.0260*	
H9A	0.60640	0.64490	0.97310	0.0240*	
H9B	0.48930	0.84220	0.97700	0.0240*	
H10A	0.48410	0.64340	0.75450	0.0280*	
H10B	0.37790	0.65200	0.91250	0.0280*	
H11A	0.18390	0.85290	0.79680	0.0320*	
H11B	0.22550	0.95280	0.90490	0.0320*	
H12A	0.21550	1.07700	0.68880	0.0330*	
H12B	0.34380	0.90240	0.59990	0.0330*	
H13A	0.45120	1.05050	0.79330	0.0280*	
H13B	0.47140	1.07860	0.62450	0.0280*	
H14A	0.73050	0.86640	0.61770	0.0230*	
H14B	0.63500	0.76060	0.60190	0.0230*	

### Atomic displacement parameters (Å^2^)

**Table:**

	*U*^11^	*U*^22^	*U*^33^	*U*^12^	*U*^13^	*U*^23^
S1	0.0207 (2)	0.0261 (2)	0.0266 (2)	−0.0094 (1)	−0.0087 (1)	0.0025 (1)
N1	0.0189 (4)	0.0140 (4)	0.0172 (4)	−0.0087 (3)	−0.0049 (3)	0.0000 (3)
N2	0.0285 (5)	0.0188 (5)	0.0241 (5)	−0.0141 (4)	−0.0079 (4)	−0.0009 (4)
N3	0.0278 (5)	0.0208 (5)	0.0241 (5)	−0.0148 (4)	−0.0090 (4)	0.0004 (4)
C1	0.0234 (5)	0.0190 (5)	0.0158 (5)	−0.0129 (4)	−0.0058 (4)	0.0023 (4)
C2	0.0203 (5)	0.0136 (5)	0.0185 (5)	−0.0089 (4)	−0.0049 (4)	−0.0011 (4)
C3	0.0177 (5)	0.0148 (5)	0.0185 (5)	−0.0092 (4)	−0.0026 (4)	−0.0023 (4)
C4	0.0234 (5)	0.0192 (5)	0.0199 (5)	−0.0112 (4)	−0.0037 (4)	0.0006 (4)
C5	0.0301 (6)	0.0187 (5)	0.0303 (6)	−0.0149 (5)	−0.0033 (5)	0.0030 (5)
C6	0.0374 (7)	0.0225 (6)	0.0336 (7)	−0.0193 (5)	−0.0051 (5)	−0.0056 (5)
C7	0.0412 (7)	0.0293 (7)	0.0226 (6)	−0.0205 (6)	−0.0059 (5)	−0.0058 (5)
C8	0.0290 (6)	0.0212 (5)	0.0176 (5)	−0.0141 (5)	−0.0033 (4)	−0.0003 (4)
C9	0.0230 (5)	0.0207 (5)	0.0161 (5)	−0.0102 (4)	−0.0024 (4)	0.0002 (4)
C10	0.0254 (6)	0.0265 (6)	0.0236 (6)	−0.0168 (5)	−0.0015 (5)	−0.0014 (5)
C11	0.0209 (5)	0.0338 (7)	0.0259 (6)	−0.0137 (5)	−0.0034 (5)	−0.0030 (5)
C12	0.0221 (6)	0.0311 (6)	0.0259 (6)	−0.0093 (5)	−0.0082 (5)	0.0009 (5)
C13	0.0248 (6)	0.0188 (5)	0.0246 (6)	−0.0085 (4)	−0.0076 (5)	0.0031 (4)
C14	0.0214 (5)	0.0182 (5)	0.0191 (5)	−0.0100 (4)	−0.0050 (4)	0.0032 (4)

### Geometric parameters (Å, º)

**Table:**

S1—C1	1.6364 (13)	C13—C14	1.5325 (18)
N1—C1	1.3357 (18)	C4—H4	0.9500
N1—C2	1.4750 (15)	C5—H5	0.9500
N1—C3	1.4359 (15)	C6—H6	0.9500
N2—N3	1.2506 (17)	C7—H7	0.9500
N2—C2	1.4786 (17)	C8—H8	0.9500
N3—C1	1.4707 (15)	C9—H9A	0.9900
C2—C9	1.5406 (19)	C9—H9B	0.9900
C2—C14	1.5356 (16)	C10—H10A	0.9900
C3—C4	1.3867 (16)	C10—H10B	0.9900
C3—C8	1.3874 (16)	C11—H11A	0.9900
C4—C5	1.3904 (18)	C11—H11B	0.9900
C5—C6	1.387 (2)	C12—H12A	0.9900
C6—C7	1.3859 (19)	C12—H12B	0.9900
C7—C8	1.392 (2)	C13—H13A	0.9900
C9—C10	1.537 (2)	C13—H13B	0.9900
C10—C11	1.532 (2)	C14—H14A	0.9900
C11—C12	1.5264 (19)	C14—H14B	0.9900
C12—C13	1.531 (2)		
			
C1—N1—C2	110.52 (10)	C6—C7—H7	120.00
C1—N1—C3	124.94 (10)	C8—C7—H7	120.00
C2—N1—C3	123.30 (11)	C3—C8—H8	120.00
N3—N2—C2	112.14 (10)	C7—C8—H8	121.00
N2—N3—C1	110.00 (11)	C2—C9—H9A	108.00
S1—C1—N1	131.05 (9)	C2—C9—H9B	108.00
S1—C1—N3	122.55 (10)	C10—C9—H9A	108.00
N1—C1—N3	106.39 (10)	C10—C9—H9B	108.00
N1—C2—N2	100.93 (10)	H9A—C9—H9B	107.00
N1—C2—C9	112.71 (9)	C9—C10—H10A	109.00
N1—C2—C14	111.24 (9)	C9—C10—H10B	109.00
N2—C2—C9	108.93 (9)	C11—C10—H10A	109.00
N2—C2—C14	107.14 (9)	C11—C10—H10B	109.00
C9—C2—C14	114.78 (11)	H10A—C10—H10B	108.00
N1—C3—C4	118.50 (10)	C10—C11—H11A	108.00
N1—C3—C8	120.00 (10)	C10—C11—H11B	108.00
C4—C3—C8	121.47 (11)	C12—C11—H11A	108.00
C3—C4—C5	119.06 (11)	C12—C11—H11B	108.00
C4—C5—C6	119.93 (12)	H11A—C11—H11B	107.00
C5—C6—C7	120.60 (13)	C11—C12—H12A	109.00
C6—C7—C8	119.93 (12)	C11—C12—H12B	109.00
C3—C8—C7	118.98 (11)	C13—C12—H12A	108.00
C2—C9—C10	115.55 (10)	C13—C12—H12B	108.00
C9—C10—C11	113.31 (11)	H12A—C12—H12B	108.00
C10—C11—C12	115.62 (12)	C12—C13—H13A	109.00
C11—C12—C13	115.09 (12)	C12—C13—H13B	109.00
C12—C13—C14	112.85 (11)	C14—C13—H13A	109.00
C2—C14—C13	114.07 (9)	C14—C13—H13B	109.00
C3—C4—H4	120.00	H13A—C13—H13B	108.00
C5—C4—H4	120.00	C2—C14—H14A	109.00
C4—C5—H5	120.00	C2—C14—H14B	109.00
C6—C5—H5	120.00	C13—C14—H14A	109.00
C5—C6—H6	120.00	C13—C14—H14B	109.00
C7—C6—H6	120.00	H14A—C14—H14B	108.00
			
C2—N1—C1—S1	179.77 (9)	N1—C2—C9—C10	−93.43 (12)
C2—N1—C1—N3	−1.29 (12)	N2—C2—C9—C10	155.42 (10)
C3—N1—C1—S1	12.16 (18)	C14—C2—C9—C10	35.32 (14)
C3—N1—C1—N3	−168.90 (10)	N1—C2—C14—C13	173.50 (11)
C1—N1—C2—N2	0.83 (12)	N2—C2—C14—C13	−77.06 (14)
C1—N1—C2—C9	−115.21 (11)	C9—C2—C14—C13	44.03 (14)
C1—N1—C2—C14	114.23 (11)	N1—C3—C4—C5	176.23 (12)
C3—N1—C2—N2	168.68 (9)	C8—C3—C4—C5	−1.9 (2)
C3—N1—C2—C9	52.64 (14)	N1—C3—C8—C7	−176.32 (13)
C3—N1—C2—C14	−77.92 (14)	C4—C3—C8—C7	1.8 (2)
C1—N1—C3—C4	67.68 (16)	C3—C4—C5—C6	0.6 (2)
C1—N1—C3—C8	−114.17 (14)	C4—C5—C6—C7	0.7 (2)
C2—N1—C3—C4	−98.41 (14)	C5—C6—C7—C8	−0.9 (2)
C2—N1—C3—C8	79.74 (15)	C6—C7—C8—C3	−0.4 (2)
C2—N2—N3—C1	−0.83 (13)	C2—C9—C10—C11	−87.16 (13)
N3—N2—C2—N1	0.05 (13)	C9—C10—C11—C12	71.74 (14)
N3—N2—C2—C9	118.86 (11)	C10—C11—C12—C13	−52.16 (16)
N3—N2—C2—C14	−116.42 (11)	C11—C12—C13—C14	70.85 (14)
N2—N3—C1—S1	−179.60 (9)	C12—C13—C14—C2	−91.21 (13)
N2—N3—C1—N1	1.35 (13)		
